# Peer Relationships and College Students’ Cooperative Tendencies: Roles of Interpersonal Trust and Social Value Orientation

**DOI:** 10.3389/fpsyg.2021.656412

**Published:** 2021-07-09

**Authors:** Gaofeng Wang, Weiwei Hu

**Affiliations:** Department of Philosophy of Science and Technology, University of Science and Technology of China, Hefei, China

**Keywords:** peer relationship, cooperative tendency, interpersonal trust, social value orientation, college students

## Abstract

The current study investigated the relationship between peer relationships and cooperative tendencies in college students, and explored the mediating role of interpersonal trust and the moderating role of social value orientation in that relationship. A questionnaire was distributed to 406 college students, and the results showed that: (1) peer relationships significantly positively predicted cooperative tendencies; (2) interpersonal trust partially mediated the relationship between peer relationships and cooperative tendencies; and (3) social value orientation moderated the relationship between peer relationships and cooperative tendencies. In particular, prosocial college students were more susceptible to peer relationships than pro-self college students. The findings of the current study indicated that college students with good peer relationships and prosocial value orientation are more likely to show the willingness to cooperate.

## Introduction

Cooperation, which is prevalent among college students, is more likely to promote positive peer relationships and goal accomplishment than competition. Previous studies on college students’ cooperative behaviors focused on scientific research cooperation and exploring factors that influence college students’ cooperative tendencies (Wang and Kong, 2019; Chen et al., 2020). However, it remains to be further explored the mechanism of cooperation tendency, which is helpful to promote college students’ cooperation in scientific research, thereby accelerating research achievements and contributing to social development.

### Peer Relationships and Cooperative Tendencies

Cooperation often takes place in the context of social interaction. It refers to a kind of joint action or way between individuals or groups to achieve a common purpose and cooperate with each other (Nowak, 2006). Therefore, it is difficult to talk about cooperative behavior without considering social relationship, which shows its importance in cooperation. Peer relationships are interpersonal relationships established and developed during social interactions among peers or individuals with similar levels of psychological development (La Greca and Harrison, 2005), and are a form of social support. At the university level, interpersonal relationships among college students are mainly peer relationships (Chai et al., 2018). Individuals interpret social situations influence their subsequent behaviors; thus, individuals’ behaviors are influenced by the social environment in which they live (Salancik and Pfeffer, 1978). Similarly, according to social exchange theory, when individuals appreciate more social support, they also tend to provide more support to others, resulting in more prosocial behaviors (Cropanzano and Mitchell, 2005). Positive peer relationships have been shown to positively predict prosocial behaviors (Bédard et al., 2014; Güroğlu et al., 2014). As cooperation is a prosocial behavior, peer relationships may have an important influence on college students’ cooperative behaviors. Having good social support facilitates better cooperation between individuals; the stronger the peer relationship, the higher the quality of cooperation in pursuit of goals shared with cooperative peers (Blair and Perry, 2019). Similarly, individuals are more likely to engage in cooperative behavior among those they consider friends (Chen et al., 2016). Consider that motivational tendency is often highly consistent with behavior (Heckhausen and Heckhausen, 2018). For example, Brooks and Rose (2008) have found that individuals with high cooperative tendency are more likely to exhibit cooperative behavior and share information with others.

### Mediating Effect of Interpersonal Trust

Research on cooperative behavior has shown that interpersonal trust positively affects cooperative tendencies among individuals (Acedo-Carmona and Gomila, 2019). Interpersonal trust reduces the cost of cooperation and is the starting point, prerequisite, and foundation of cooperative relationships (Malti et al., 2016). Interpersonal trust is a measure of individual relationships. When peer trust is high, relationships are closer and cooperative tendencies are more significant. However, when peer trust is low, relationships are more distant, resulting in less significant cooperate tendencies (Wang and Chen, 2011). When team members are trustworthy or can exclude individuals who may defect, “piggyback” off others, or be uncooperative, the teamwork will be better quality (Yoeli et al., 2013). Accordingly, it can be posited that interpersonal trust positively affects cooperative tendencies. The internal working model of social support suggests that individuals who gain trust in peer relationships also develop a sense of trust in others. This, in turn, affects their interpersonal interactions and increases the likelihood they will respond positively to others and develop prosocial behaviors (Moreira et al., 2003; Zhao et al., 2017). Furthermore, when individuals perceive social rejection and have more distant peer relationships, they have significantly lower levels of trust than socially accepted individuals (Xu et al., 2017), suggesting a positive correlation between peer relationships and interpersonal trust. Thus, it can be inferred that, among college students, those with good peer relationships will experience more interpersonal trust, to some extent.

### Moderating Effect of Social Value Orientation

Based on the perspective of individual-context interaction (Lerner et al., 2006), individual behavior is formed and developed in the interaction between the individual and the environment. Not all individuals will have a higher level of trust or a greater tendency to cooperate, and personality traits may play a moderating role (Bartlett and DeSteno, 2006). Previous studies have found that social value orientation, as a personality trait, affects individuals’ trust behavior. Specifically, different from pro-self individuals, prosocial individuals gave higher rewards to trustees who were more trustworthy (Kanagaretnam et al., 2009; Derks et al., 2014). Social value orientation refers to individuals’ tendencies toward distributing benefits to themselves, others, or groups, when facing social difficulties (Yuan et al., 2014). Individuals can have either a prosocial or pro-self value orientation. Prosocial individuals generally seek to equalize or maximize group interests, while pro-self individuals generally prioritize their own interests (Bogaert et al., 2008). Compared with pro-self individuals, prosocial individuals have been shown to be more likely to trust others, are more likely to trust others (Derks et al., 2014, 2015), and have more spontaneous trust behaviors (van den Bos et al., 2009). Research has also found that personality traits can indirectly influence cyber interpersonal trust through cyber social support (Ding and Sheng, 2005). Thus, social value orientation may indirectly influence interpersonal trust through peer relationships. As a subjective tendency, social value orientation differs among college students, and pro-self individuals generally show less prosocial behavior. Therefore, peer relationships are more likely to influence interpersonal trust in college students with a pro-self orientation than in those with a prosocial value orientation.

Early research suggests that the propensity for interpersonal trust is high in prosocial individuals and low in pro-individuals (Liebrand et al., 1986). In studies of the prisoner’s dilemma, prosocial individuals were likely to exhibit cooperative behavior, whereas pro-self individuals tended to choose betrayal (Emonds et al., 2011, 2014). Prosocial people value harmony and fairness, and are therefore more likely to cooperate with others, while pro-self individuals are more likely to exploit others for personal gain than to cooperate (de Dreu, 2010). Wang and Chen (2011) found that when prosocial individuals are punished, their interpersonal trust decreases, which negatively affects their level of cooperation. Thus, interpersonal trust has a greater influence on the cooperation tendencies of prosocial individuals.

As previous studies have often neglected the critical role of self-regulation, the current study aimed to test the possible moderating role of social value orientation in the relationship between peer relationships and cooperative tendencies. Behavioral dynamics theory posits that individuals’ behaviors are influenced by a combination of personality traits and the social environment, thereby suggesting that peer relationships and social value orientations could significantly influence individuals’ cooperative behaviors. Using the Chicken Game, Yuan et al. (2014) examined the cooperative and conflict behaviors toward friends or strangers of individuals with different social value orientations. They found that prosocial individuals cooperated more with friends than strangers, while pro-self individuals showed no differences in their cooperative behaviors toward friends and strangers. Thus, peer relationships have more influence on prosocial individuals’ cooperative tendencies, compared with pro-self individuals. Based on this, it could be inferred that social value orientation might help individuals to self-regulate when peer relationship affinity is insufficient in the cooperation process, thus influencing cooperative behavior.

### Present Study

The current study aimed to examine the effects of peer relationships, interpersonal trust, and social value orientation on college students’ cooperative tendencies. Specifically, two research questions were explored. First, does interpersonal trust play a mediating role in the process by which peer relationships influence cooperative tendencies? Second, do social value orientations moderate the mediating processes by which peer relationships affect cooperative tendencies? This study presented five hypotheses as follows:

*Hypothesis 1*: Peer relationships positively influence cooperative tendencies.

*Hypothesis 2*: Interpersonal trust plays a mediating role in the process by which peer relationships influence cooperative orientation.

*Hypothesis 3*: Social value orientation plays a moderating role in the first half of the intermediary path of “peer relationships → interpersonal trust → cooperative tendencies.”

*Hypothesis 4*: Social value orientation plays a moderating role in the second half of the intermediary path of “peer relationships → interpersonal trust → cooperative tendencies.”

*Hypothesis 5*: Social value orientation has a moderating role in the direct pathway by which peer relationships influence cooperative tendencies.

**Figure 1:**
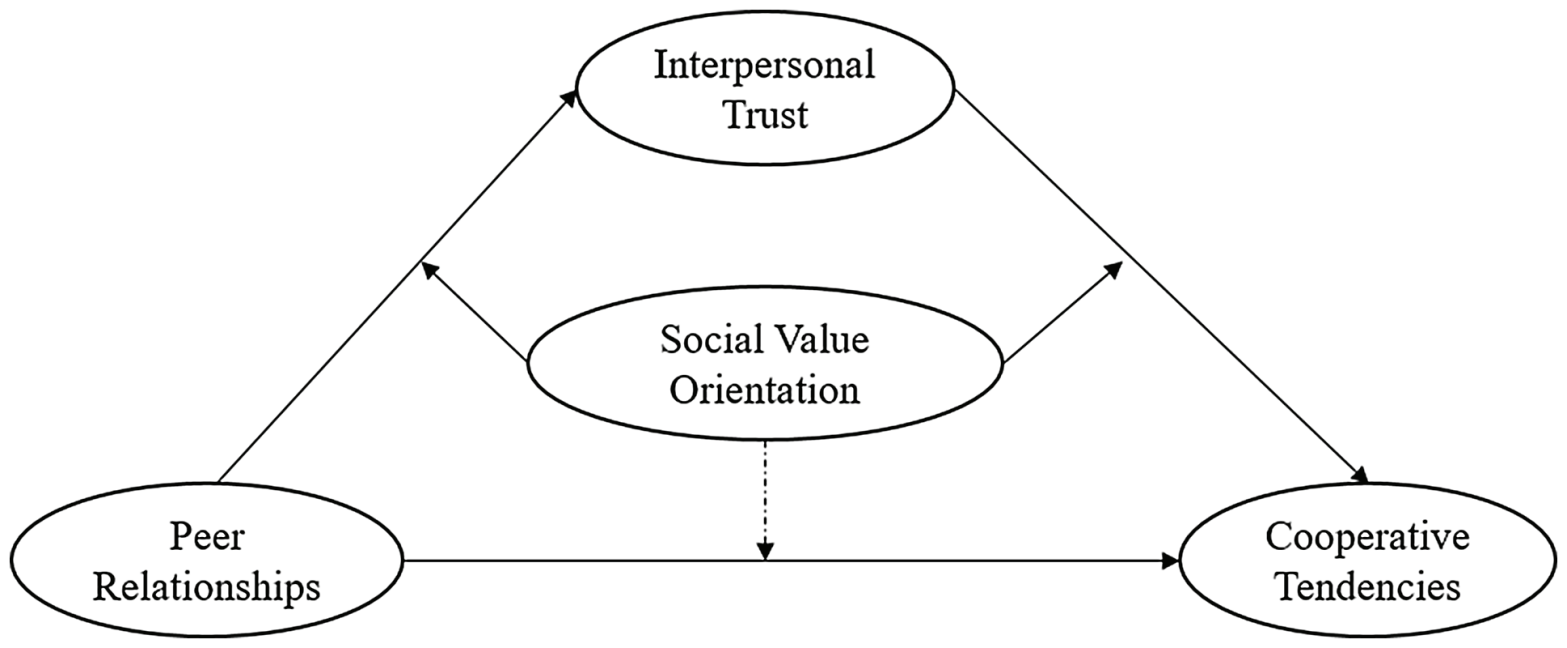
Diagram of the hypothesized moderated mediation model.

Based on the above hypotheses, this study constructed a moderated mediation model for the relationship between peer relationship and cooperative tendency (see Figure 1).

## Materials and Methods

### Participants

Through the stratified sampling method, a questionnaire survey was conducted in several universities in a province in China. A total of 436 questionnaires were distributed, with 406 valid questionnaires returned (recovery rate: 93.12%). There were 282 men and 124 women, aged between 18 and 24 years, with an average age of 20.41 years (*SD* = 1.06). Participants in the present study involved undergraduates and postgraduates who all have research experiences. In order to ensure high statistical efficiency, G*Power soft pieces (Faul et al., 2009) were used to calculate the sample size. According to Cohen (1988), the medium effect size was set (*r* = 0.02). A minimum of 389 samples were required for this study, providing a statistical power of 98% (1 − *β*). The Ethics Committee of University of Science and Technology of China approved this study, in accordance with the ethical principles of Declaration of Helsinki. All participated voluntarily and received some remuneration or an equivalent gift upon completing the questionnaire.

### Procedures

This study was measured using a questionnaire collected by professionally trained graduate students. After receiving an explanation of the requirements, the participants completed a questionnaire during class time/with their class. During the process of completing the questionnaire, any questions that the participants did not understand could be put forward to the experimenters. The questionnaire was filled in anonymously and collected immediately upon completion, which took about 10 min.

### Measurement Tools

#### Peer Relationships

The Peer Relationship Inventory, as revised by Zou (1998), was used to measure individuals’ self-perceptions during interactions with others. It has 30 items in two dimensions (peer acceptance and fear of inferiority). Responses were rated on a four-point Likert scale, ranging from 1 = “completely disagree” to 4 = “completely agree.” All items except 1, 3, 4, 7, 11, and 17 were reverse scored, with higher total scores on the subdimensions indicating higher self-perceived peer acceptance, better peer relationships, and greater popularity in the classroom. In this study, the internal consistency coefficient of this scale was 0.92.

#### Interpersonal Trust

Based on Rotter (1967), the Chinese Interpersonal Trust Scale revised by Xu (2010) was used to measure trust in different individuals (e.g., parents and teachers). The scale includes 25 items across two dimensions: trust in direct peers and trust in non-direct peers. Responses were rated on a five-point Likert scale, ranging from 1 = “completely disagree” to 5 = “completely agree.” Items 1–5, 7, 9, 10, 11, 13, 15, 19, and 24 were reverse scored; higher overall scores indicated higher levels of interpersonal trust. In this study, the internal consistency coefficients for the two dimensions were 0.88 and 0.82, respectively, and the overall internal consistency coefficient was 0.85.

#### Social Value Orientation

The Triple-dominance Matrix questionnaire was to measure social value orientation. Wang and Ji (2009), Wang and Chen (2011) used this method to measure individual value orientation for many times, which proved that this measurement method has good internal consistency and retest reliability (de Dreu and Mccusker, 1997). This tool has 12 items, each consisting of three choices, in which participants are asked to decide on three different distributions between themselves and another person. The three choices for each item followed the following pattern: In option 1, participants score the highest of the three choices; in option 2, the score difference between the participants and others is the largest of the three choices; and in option 3, the sum of the scores of the participants and others is the highest of the three choices. The three choices are randomly disrupted when actually filled out. A preference for option 1 indicates that the respondent has an individualistic orientation, option 2 indicates a competitive orientation, and option 3 indicates a cooperative orientation. Following de Dreu and McCusker (1997), participants who responded consistently in 7 out of 12 decisions were classified into one of two categories: pro-self orientation (in which individualistic orientation and competitive orientation were combined) and cooperative orientation. In the current study, 348 participants were classified as having a cooperative orientation, 38 as having pro-self orientation (including 11 with individualistic orientation and 27 with competitive value orientation), and 20 people were excluded due to ambiguous choices.

#### Cooperative Tendencies

The cooperative tendency rating subscale of the Cooperative and Competitive Personality Tendencies Scale by Xie et al. (2006) was used to measure individual degrees of cooperative tendencies. The subscale has 16 items and three subdimensions: inclusiveness, reciprocity, and willingness to cooperate. Responses were rated on a five-point Likert scale, ranging from 1 = “completely disagree” to 5 = “completely agree.” Higher scores indicate higher cooperative tendencies. In this study, the internal consistency coefficients for the three subdimensions were 0.82, 0.86, and 0.80, respectively, and 0.85 for the total subscale.

### Statistical Analyses

After eliminating invalid questionnaires (e.g., missing some answers and regular answers), SPSS 22.0 was used for hierarchical regression to test for moderated mediation effects. Model 1 and Model 4 of the PROCESS macro program (download address: http://www.Afhayes.com/) by Hayes (2014) were used to examine the moderated mediation model. AMOS 22.0 was used to test the integrated model.

## Results

### Common Method Bias Control and Test

Since all data were based on participant self-report, the results were susceptible to common method bias. According to Zhou and Long (2004), controls, such as the use of participant anonymity and reverse scoring of some items, were applied during the procedure. Harman’s single-factor test was adopted to test for common method bias. The results showed that the 22 eigenvalues obtained both without and after rotation were all greater than 1. Additionally, 19.35% of the variance was explained by the first factor without rotation, and 9.03% was explained with rotation, which were both less than the critical value of 40%, indicating there was no significant common method bias in this study.

### Means, Standard Deviations, and Correlation Matrices

Table 1 shows the means, standard deviations, and correlation matrices of the study variables. Peer relationships were significantly and positively correlated with cooperative tendencies and interpersonal trust, interpersonal trust was significantly and positively correlated with cooperative tendencies, and social value orientation was not significantly correlated with cooperative tendencies. Regarding demographic variables, interpersonal trust was significantly correlated with gender, and an independent sample *t*-test found that women had higher interpersonal trust scores than men [*t*_(404)_ = −2.27, *p <* 0.05]. To examine the correlations between variables in depth, a moderated mediation model was tested.

**Table 1:** Means, standard deviations, and correlation matrices of variables.

S. No.		*M*	*SD*	1	2	3	4	5	6
1.	Gender^a^	–	–	1					
2.	Age	24.02	2.55	−0.08	1				
3.	Peer relationships	3.02	0.45	0.05	0.05	1			
4.	Interpersonal trust	3.85	0.27	0.11^*^	−0.03	0.35^**^	1		
5.	SOV^b^	–	–	0.05	−0.09	−0.01	0.07	1	
6.	Cooperative tendencies	3.76	0.50	0.05	0.06	0.31^**^	0.17^**^	0.01	1

*SOV, social value orientation;*

a *is a dummy variable; 0=female; 1=male;*

b *is a dummy variable, 0=self-orientation, 1=social orientation; The following is the same*.

* *p < 0.05;*

** *p < 0.01.*

### Moderated Mediation Model Testing

After standardizing the data, it was found that the variance inflation factors of all predictor variables were below 1; thus, there was no problem with multicollinearity. According to Wen and Ye (2014), examining a moderated mediation model requires testing the parameters of three regression equations: (1) Equation 1 estimates the moderating effect of the moderating variables on the relationship between the independent and dependent variables; (2) Equation 2 estimates the moderating effect of the moderating variables on the relationship between the independent and mediator variables; and (3) Equation 3 estimates the moderating effect of the moderating variables on the relationship between the mediator and dependent variables, and the moderating effect of the independent variable on the residual effect of the dependent variable (Table 2). As shown in Table 2, for Equation 1, peer relationships positively predicted cooperative tendencies (*β* = 0.32, *p <* 0.001); however, the interaction between peer relationships and social value orientation did not significantly predict cooperative tendencies (*β =* 0.03, *p >* 0.05). For Equations 2 and 3, the interaction between peer relationships and social value orientation significantly predicted interpersonal trust (*β =* −0.12, *p* < 0.01), while the main effect of interpersonal trust on cooperative tendencies was significant (*β =* 0.12, *p <* 0.05). Peer relationships had a significant main effect on interpersonal trust (*β =* 0.34, *p <* 0.0001), while the interaction between interpersonal trust and social value orientation did not significantly predict cooperative tendencies (*β =* 0.07, *p >* 0.05). This suggested that peer relationships, interpersonal trust, social value orientation, and cooperative tendencies form a moderated mediation effect model, with interpersonal trust playing a mediating role in the relationship between peer relationships and cooperative tendencies, with the mediation effects explaining 4.2% of the total effects. Furthermore, social value orientation played a moderating role in the first half of the model’s path.

**Table 2:** Moderated mediation effect model.

	Equation 1 (*Y*: Cooperative Tendencies)				Equation 2 (*M*: Interpersonal Trust)				Equation 3 (*Y*: Cooperative Tendencies)			
	*B*	*SE*	*β*	95% CI	*B*	*SE*	*B*	95% CI	*B*	*SE*	*β*	95% CI
Peer relationships *X*	0.31	0.05	0.32^***^	[0.22, 0.41]	0.34	0.05	0.34^***^	[0.25, 0.43]	0.28	0.05	0.29^***^	[0.18, 0.39]
*SOV U*	0.01	0.05	0.05	[−0.09, 0.10]	0.08	0.05	0.07	[−0.02, 0.17]	−0.01	0.05	−0.01	[−0.10, 0.09]
*XU*	0.03	0.04	0.03	[−0.06, 0.12]	−0.11	0.04	−0.12^**^	[−0.20, −0.02]	0.06	0.06	0.06	[−0.05, 0.17]
Interpersonal trust *M*									0.12	0.05	0.12^*^	[0.04, 0.15]
*MU*									−0.03	0.06	−0.04	[−0.14, 0.08]
*R*^2^	0.10^***^				0.14^***^				0.11^***^			
*F*	14.31^***^				21.01^***^				9.08^***^			

* *p < 0.05;*

** *p < 0.01;*

*** *p < 0.001.*

**Figure 2:**
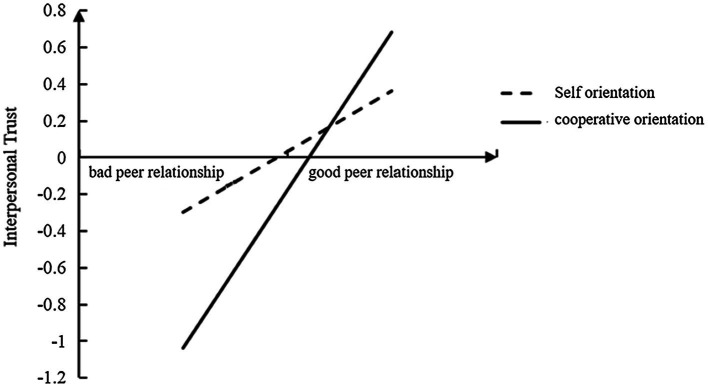
The moderating role of social value orientation in peer relationships affecting interpersonal trust.

To better explain the moderated mediation model, peer relationships were divided into “bad” and “good” groups, according to the mean plus or minus one standard deviation, and a simple slope test was used to investigate the influence of social value orientation in peer relationships on interpersonal trust. The specific moderating effects are presented in Figure 2. The results showed that as peer relationships improved, interpersonal trust increased. However, there were differences between individuals with different social value orientations, with a significantly higher upward trend in the level of interpersonal trust (*b*_simple_ = 0.86, *t =* 6.65, *p <* 0.001) for cooperative orientation than for pro-self orientation (*b*_simple_ = 0.32, *t =* 7.05, *p <* 0.001). This suggested that cooperative orientation can facilitate the positive impact of peer relationships on interpersonal trust.

**Figure 3:**
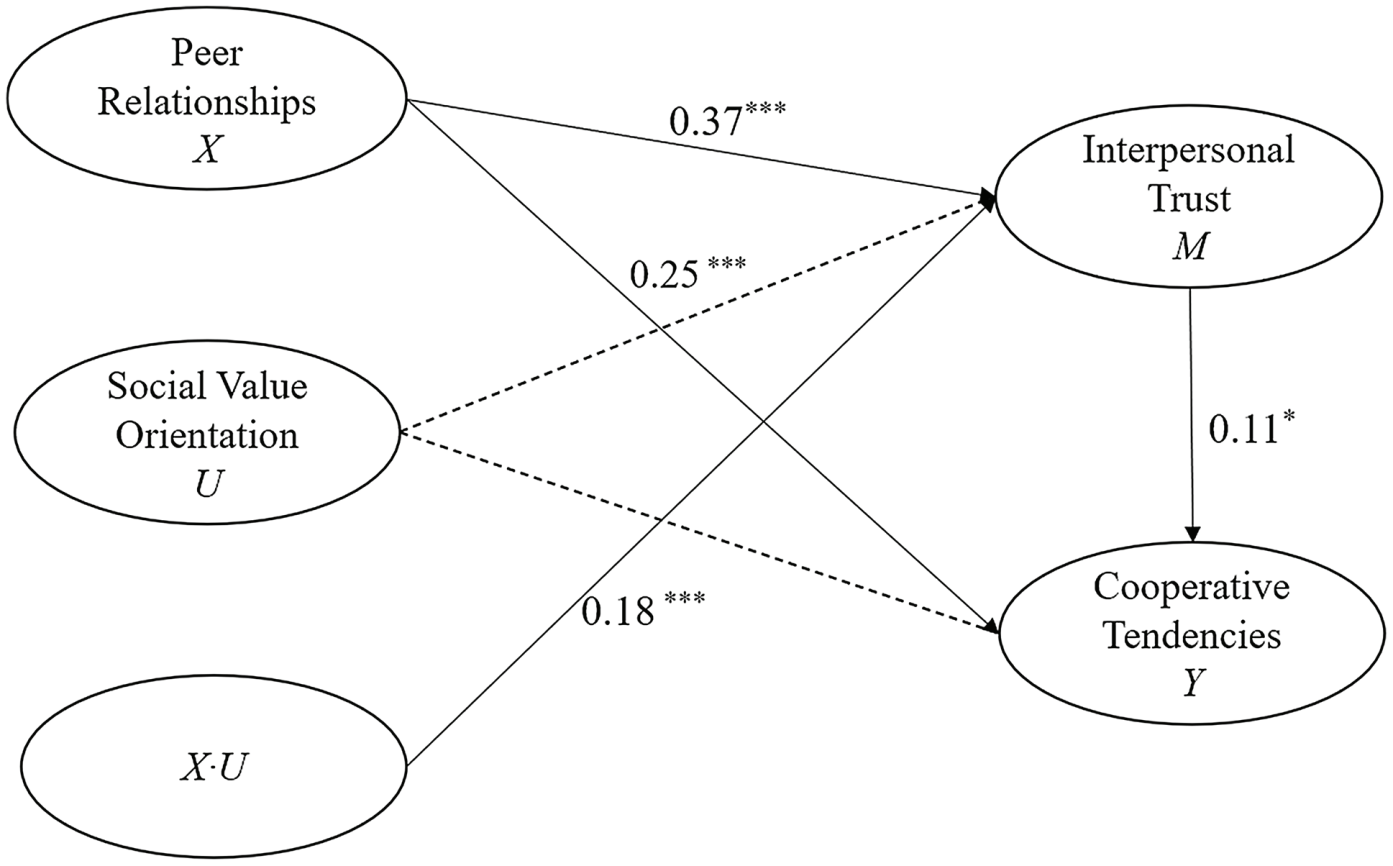
The path coefficient diagram. ^*^p < 0.05; ^***^p < 0.001.

Finally, the integration model was further tested using AMOS 22.0, and its path coefficient diagram is shown in Figure 3. The results showed good model fit (*χ*^2^/df = 3.84, CFI = 0.94, NFI = 0.93, GFI = 0.92, and RMSEA = 0.05). Peer relationships significantly positively predicted cooperative tendencies (*γ* = 0.25, *p <* 0.001). Additionally, peer relationships significantly positively predicted interpersonal trust (*γ* = 0.37, *p <* 0.001), and interpersonal trust significantly positively predicted cooperative tendencies (*γ* = 0.11, *p <* 0.05). The interaction between social value orientation and peer relationships significantly positively influenced interpersonal trust (*γ* = 0.18, *p <* 0.001); however, the interaction between social value orientation and interpersonal trust did not significantly influence cooperative tendencies (*γ* = 0.01, *p =* 0.652). This suggested that interpersonal trust has a partially mediating role in the process by which peer relationships influence cooperative tendencies, and that the moderating role of social value orientation occurs only in the first half of the path, with the moderating mediation model established.

## Discussion

The current study found that peer relationships positively predicted college students’ cooperate tendencies. Moreover, it was found that peer relationship affected cooperative tendency through interpersonal trust, and social value orientation moderated the relationship between peer relationship and interpersonal trust, which supported Hypotheses 1 to 3. Fehr et al. (2008) argued that the reason peers or friends are more likely to cooperate with each other is related to the motivation to cooperate. Specifically, as individuals expect to gain future benefits from their interaction partners, those who do not have peer relationships with themselves, the individual’s lack of such expected, and therefore less likely to engage in cooperative behavior. This suggests that college students should focus not only on their studies but also on interpersonal interactions with classmates. Considering that high-quality friendships are the foundation of good peer relationship (Zhang et al., 2011; Chai et al., 2018), college students should pay more attention to the quality of peer relationship. Maintaining good peer relationships can increase cooperative tendencies and create more opportunities for research collaboration.

### Mediating Role of Interpersonal Trust

The findings of the current study indicated that interpersonal trust plays a partial mediating role in the process by which peer relationships influence cooperative tendencies, and this mediation model supported the internal working model of social support. Good peer relationships, as a favorable condition of social support, can help individuals form good character traits and interpersonal trust (Sun et al., 2015), shorten the psychological distance between individuals, and thus make people more cooperative with their peers and more willing to pursue goals together. Therefore, cooperative tendencies between peers who trust each other are generally greater than those of individuals who have not built trust. This suggests that college students should fully trust their peers when cooperating, in order to better accomplish common goals. However, interpersonal trust is not a necessary condition for peer cooperation, as cooperation is possible even without trust (Kulms and Kopp, 2018).

The current study does not rule out the possibility that there are other mediators by which peer relationships can change the behavior by influencing the psychological distance between individuals. For example, individuals who are emotionally dependent are more likely to cooperate than those who are rationally dependent (Levine et al., 2018). It has also been found that as individuals age, whether they show poor prosocial behavior toward peers depends on peer relationships, with a greater preference for friends (Güroğlu et al., 2014). Therefore, interpersonal trust, as a bridge between peer relationships and cooperative tendencies, may be an important factor for enhancing the psychological distance between peers and interpersonal trust is an element of cooperative tendencies among college students. Whether there are other mediating variables between peer relationships and cooperative tendencies will be explored in subsequent studies.

### Moderating Role of Social Value Orientation

In this study, social value orientation only moderates the relationship between peer relationship and interpersonal trust, thus supporting Hypothesis 3. Compared with the pro-self individuals, good peer relationship can significantly predict the interpersonal trust of prosocial individuals. This is consistent with previous studies that found that prosocial individuals are more likely to trust others (Derks et al., 2014). At the same time, previous studies have found that prosocial individuals are more susceptible to context factors and give priority to common interests. This may promote prosocial individuals to care more about good interpersonal relationships and thus have a higher level of trust (Bogaert et al., 2008; Fiedler et al., 2013). In contrast, the pro-self individual emphasizes self-interest maximization and lacks sensitivity to others’ evaluation and interpersonal relationship (Utz et al., 2004; Weber et al., 2004).

This study found that social value orientation did not mediate the relationship between interpersonal trust and cooperative tendency. Previous results have shown that prosocial people are more likely to cooperate than people who are personally self (Mischkowski and Glöckner, 2016). However, prosocial people do not always show cooperative tendencies, and it may be in certain situations that prosocial people cooperate. For example, a study using resource dilemmas associated with water scarcity found that prosocial people stop their water-saving behavior when their behavior fails to achieve the desired goal due to uncontrollable factors (i.e., reduced water resources; Brucks and van Lange, 2007). In addition, with the increase in interpersonal trust, individuals tend to choose common goals or engage in behaviors that generate common interests, and prosocial behaviors also increase (Zhao and Zhang, 2013). So, it is possible that whether an individual is prosocial or personally self, when individuals perceive interpersonal trust, they tend to cooperate.

## Conclusion

### Significance

The current study has certain theoretical implications for the promotion of research cooperation among college students. From a peer relationship perspective, this study explored the influence of interpersonal trust and social value orientation on college students’ cooperative tendencies. On the one hand, it expanded the previous research on the influence of peer relationship on individual prosocial behavior and supported the social exchange theory, pointing out that situational factors can affect individual prosocial behavior, and peer relationship and social support play a crucial role in promoting cooperation. On the other hand, this study further explored the effect path of peer relationship on cooperative tendency, revealed that peer relationship promotes win-win cooperation through enhancing trust perception, and focused on the moderating role of personality traits in this process.

Additionally, the current study also has practical implications for promoting research cooperation among college students. Firstly, college students are in the middle period between adulthood and entering society, and peer relationships play an important role in the development of college students. In the process of research cooperation, if college students respect and trust partners more, it will promote mutual benefit and win-win results. Secondly, teachers can pay attention to and deal with the problem of peer communication to help college students form prosocial values. Finally, the organization and management of the research team should not ignore the peer relationship between individuals and provide good social support. All of these can improve the interpersonal trust and mutual benefit of individuals, and greatly promote good scientific research cooperation.

### Limitations and Future Directions

The current study had the following limitations that should be addressed in subsequent studies. First of all, this study posited that there may be other mediating variables between peer relationships and cooperative tendencies, such as perceived responsibility, group identification, and individual personality traits and feelings of wear (Olson and Janes, 2002; Fehr and Rockenbach, 2003; Chen, 2016). Therefore, subsequent studies should control experimental conditions and compare the effects of different combinations of personality traits, gender, age, and family of origin on cooperative tendencies among college students. In addition, future research can further explore the longitudinal influence of peer relationship on cooperative tendency and behavior development, and provide causal evidence for revealing and promoting cooperative behavior (Zhou et al., 2020).

### Summary

The main findings of the current study were as follows: (1) peer relationships significantly and positively predicted cooperative tendencies; (2) interpersonal trust partially mediated the process by which peer relationships influence cooperative tendencies; and (3) the moderating effect of social value orientation occurred only in the first half of the intermediary path of “peer relationships → interpersonal trust → cooperative tendencies.”

## Data Availability Statement

The raw data supporting the conclusions of this article will be made available by the authors, without undue reservation.

## Ethics Statement

The studies involving human participants were reviewed and approved by University of Science and Technology of China. The patients/participants provided their written informed consent to participate in this study.

## Author Contributions

GW conceived and designed the study. GW and WH performed the study and wrote the paper. All authors contributed to the article and approved the submitted version.

### Conflict of Interest

The authors declare that the research was conducted in the absence of any commercial or financial relationships that could be construed as a potential conflict of interest.
